# Effect of selection bias on two sample summary data based Mendelian randomization

**DOI:** 10.1038/s41598-021-87219-6

**Published:** 2021-04-07

**Authors:** Kai Wang, Shizhong Han

**Affiliations:** 1 Department of Biostatistics, The University of Iowa, Iowa City, 52242 USA; 2 Lieber Institute for Brain Development, Johns Hopkins School of Medicine, Baltimore, 21205 USA; 3 Department of Psychiatry and Behavioral Sciences, Johns Hopkins School of Medicine, Baltimore, 21205 USA

**Keywords:** Genetics, Gene expression, Genetic association study

## Abstract

Mendelian randomization (MR) is becoming more and more popular for inferring causal relationship between an exposure and a trait. Typically, instrument SNPs are selected from an exposure GWAS based on their summary statistics and the same summary statistics on the selected SNPs are used for subsequent analyses. However, this practice suffers from selection bias and can invalidate MR methods, as showcased via two popular methods: the summary data-based MR (SMR) method and the two-sample MR Steiger method. The SMR method is conservative while the MR Steiger method can be either conservative or liberal. A simple and yet more powerful alternative to SMR is proposed.

## Introduction

As a feasible alternative to expensive and sometimes impossible randomized clinical trials, Mendelian randomization (MR) is becoming more and more popular for inferring causal relationship between an exposure and a trait^1–3^. Summary data-based two-sample MR methods often take the following two steps:

Step 1: Obtain instruments (typically SNPs) from exposure GWAS (Genome-Wide Association Study) that are significant at genome-wide level (typically $p<5\times 10^{-8}$);
Step 2: Investigate the causal relationship between the exposure and the trait, using the summary exposure GWAS statistics at the selected SNPs and a trait GWAS. The summary exposure GWAS statistics are those used in Step 1 for SNP selection.

One appealing feature of these methods is that they only rely on summary statistics on the exposure GWAS and the trait GWAS. Individual-level data are not needed.

The inference validity of this two-step approach is affected by selection bias. When conducting causal inference in Step 2 with respect to the SNPs selected in Step 1, the summary statistics from the exposure GWAS can not be regarded as random samples for the true population association strength^4–6^. Treating them as random samples leads to over-estimation of the effect size of these SNPs on the exposure. Association strength in a random sample is often much weaker, a phenomenon commonly seen in studies aimed at replicating previous findings. This selection bias has been noted in the literature^6,7^. But its effect on hypothesis testing related to two sample summary data based Mendelian randomization is largely unknown.

Two popular MR methods, the summary data-based MR method^2^ and the two-sample MR Steiger method^1^, are considered. For the summary data-based MR method, the most significant SNP (instead of several SNPs) from a gene is selected as the instrument from the exposure GWAS. For the two-sample MR Steiger method, a SNP significantly associated with both the exposure GWAS and the trait GWAS is selected. The genotype score (0, 1, or 2) at this SNP is denoted by *g*. The exposure level is denoted by *x* and the trait value is denoted by *y*. The Wald statistic on chi-square scale for testing the association between the SNP and the exposure is denoted by $W_{gx}$. Its value is supposed to be large because it satisfies the selection criterion used in Step 1. For instance, when the selection criterion is $p<5\times 10^{-8}$, there must be $W_{gx}>29.71679$. The Wald statistic for testing the association between the SNP and the trait is denoted by $W_{gy}$, which is independent of $W_{gx}$.

## Results

### Summary data-based MR

Summary data-based MR^2^ (SMR) is a popular MR method for inferring causality between *x* and *y*. Its null is $H_0: b_{xy}=0$, where $b_{xy}$ is the true regression coefficient for *x* with *y* the response. The two-stage least square (2SLS) estimate of $b_{xy}$ is

1 $$\begin{aligned} \hat{b}_{xy} = \frac{\hat{b}_{gy}}{\hat{b}_{gx}}, \end{aligned}$$

where $\hat{b}_{gx}$ is the least square estimate of $b_{gx}$, the regression coefficient for *g* with *x* the response, and $\hat{b}_{gy}$ is the least square estimate of $b_{gy}$, the regression coefficient for *g* with *y* the response. $\hat{b}_{xy}$ is also known as the Wald ratio^5^. Causal relationship between exposure *x* and *y* exists if the following test statistic is significant^2^:

$$\begin{aligned} T_\text {SMR} = \frac{W_{gx}W_{gy}}{W_{gx}+W_{gy}}, \end{aligned}$$

where $W_{gx} = [\hat{b}_{gx} / SE({\hat{b}}_{gx})]^2$ and $W_{gy} = [{\hat{b}}_{gy} / SE({\hat{b}}_{gy})]^2$ are Wald statistics on chi-square scale. The null distribution of $T_\text {SMR}$ is approximated by 1-df chi-square using the Delta method^2^.

**Figure 1:**
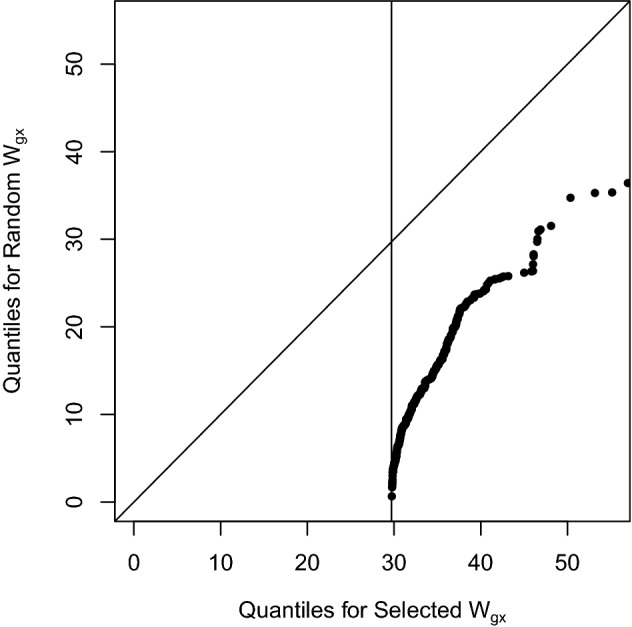
Quantile-quantile plot for selected $\{W_{gx}\}$ (322 out of 10,000) and 322 random $\{W_{gy}\}$. The distribution of selected $\{W_{gx}\}$ is different from the distribution of random $\{W_{gy}\}$ as shown by the deviation of the points from the 45° line. The vertical line indicates the selection threshold $W_{gx}\ge 29.71679$ which corresponds to genome-wide significance level $5\times 10^{-8}$.

There are several issues with statistic $T_\text {SMR}$. The derivation of its null distribution assumes that ${\hat{b}}_{gx}$ is a consistent estimator of $b_{gx}$ and (asymptotically) follows a normal distribution (^2^, Online Methods). However, these two conditions do not hold. If the significance level used in Step 1 is $5\times 10^{-8}$, there must be $W_{gx}\ge 29.71679$ which implies $|\hat{b}_{gx}| \ge \sqrt{29.71679}\times SE(\hat{b}_{gx})$. As a result, the distribution of $\hat{b}_{gx}$ is not (asymptotically) normal and ${\hat{b}}_{gx}$ is not a consistent estimator of $b_{gx}$. To numerically demonstrate this point, 10,000 random samples of $W_{gx}$ are generated from a 1-df chi-square with a large non-centrality 13 (to make sure there are reasonable number of $\{W_{gx}\}$). Among them, 322 are significant at genome-wide significant level $5\times 10^{-8}$. The quantile-quantile plot of these selected $\{W_{gx}\}$ against 322 random samples $\{W_{gy}\}$ from 1-df chi-square with non-centrality 13 is shown in Fig. 1. The distribution of $\{W_{gx}: W_{gx}\ge 29.71679\}$ is clearly different from the distribution of $\{W_{gx}\}$.

The applicability of the Delta method to approximating the distribution of $T_\text {SMR}$ is in doubt even in the absence of the selection process imposed on $W_{gx}$. Approximating the null distribution of $T_\text {SMR}$ by a 1-df chi-square is equivalent to approximating the null distribution of ${\hat{b}}_{xy}$ by a normal distribution. However, according to Eq. (1), ${\hat{b}}_{xy}$ is a ratio of two normals. In general, the distribution of the ratio of two normal variables can not be approximated by a normal as it can take a variety of shapes such as bimodal, unimodal, or asymmetric^8^. It is known that if $b_{gx}$ and $b_{gy}$ are both equal to 0, the distribution of ${\hat{b}}_{xy}$ would be a Cauchy, a fat-tailed distribution whose mean and variance do not exist. For the case $b_{gx}\not =0$ and $b_{gy}\not =0$, the distribution of ${\hat{b}}_{xy}$ can be approximated by a normal only in certain intervals^8^.

**Figure 2:**
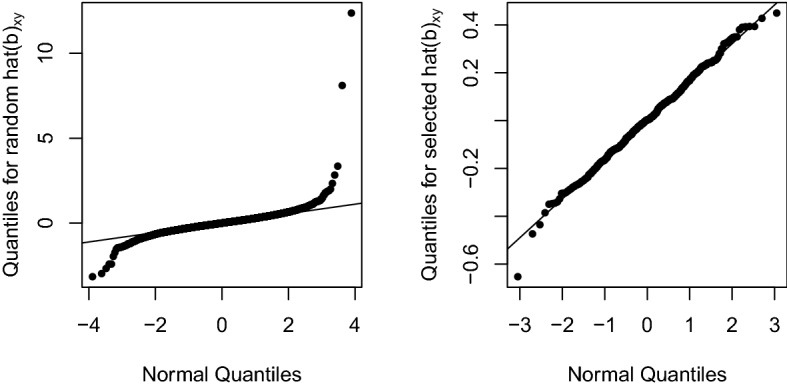
Normal quantile plot for 10,000 ${\hat{b}}_{xy} = {\hat{b}}_{gy}/{\hat{b}}_{gx}$ generated under $b_{gx}=\sqrt{13}$ and $b_{gy}=0$. The distribution of $\{{\hat{b}}_{xy}\}$ appears to be fat-tailed compared to a normal (left panel). The distribution of $\{{\hat{b}}_{xy}: {\hat{b}}_{gx} \text { is significant}\}$ (436 out of 10,000) seems to be a normal (right panel) due to selection imposed on ${\hat{b}}_{gx}$. See the text for explanation.

For the case $b_{gy}=0$, to our best knowledge, there are no known theoretical results regarding whether the distribution of ${\hat{b}}_{xy}$ can be approximated by a normal. The only thing we are sure about is that the distribution of ${\hat{b}}_{xy}$ is symmetric because the distribution of $-{\hat{b}}_{xy}=(-{\hat{b}}_{gy})/{\hat{b}}_{gx}$ is the same as the distribution of ${\hat{b}}_{xy}$. A numerical example is used to examine the distribution of ${\hat{b}}_{xy}$. Ten thousand random ${\hat{b}}_{gx}$’s are generated from $N(\sqrt{13}, 1)$ and 10,000 random ${\hat{b}}_{gy}$ are generated from *N*(0, 1). A normal quantile plot of ${\hat{b}}_{gy}/{\hat{b}}_{gx}$ is generated using the qqnorm and qqline functions in R with their default settings and is shown in Fig. 2 (left panel). Similar to a Cauchy distribution, the distribution of ${\hat{b}}_{xy}={\hat{b}}_{gy}/{\hat{b}}_{gx}$ is apparently fat-tailed compared to a normal: the lower end is more negative while the upper end is more positive.

A normal quantile plot is also generated for $\{{\hat{b}}_{xy}: \hat{W}_{gx}\ge 29.71679\}$ and is shown in the right panel of Fig. 2. It may be surprising that the distribution of $\{{\hat{b}}_{xy}: W_{gx}\ge 29.71679\}$ appears to be a normal. The reason of this phenomenon is that the range of ${\hat{b}}_{gx}$ is greatly reduced under the selection criterion. According to Eq. (1), ${\hat{b}}_{xy}$ is roughly proportional to ${\hat{b}}_{gy}$ with high probability.

A more general argument that the approximating distribution of $T_\text {SMR}$ is not 1-df chi-square is the following. Since

$$\begin{aligned} T_\text {SMR} = W_{gy}\cdot \frac{1}{1+W_{gy}/W_{gx}}, \end{aligned}$$

there is $T_\text {SMR}< W_{gy}$ regardless of the distribution of $W_{gx}$. That is, $T_\text {SMR}$ is always dominated by $W_{gy}$. Similarly, $T_\text {SMR}$ is always dominated by $W_{gx}$. Therefore, $T_\text {SMR}<\min \{W_{gx}, W_{gy}\}$. Since $W_{gx}$ and $W_{gy}$ approximately follow independent 1-df chi-square distributions, the approximating distribution of $\min \{W_{gx}, W_{gy}\}$ can not be 1-df chi-square. Neither the approximate distribution of $T_\text {SMR}$. Using a 1-df chi-square distribution for $T_\text {SMR}$ results in a conservative test.

**Figure 3:**
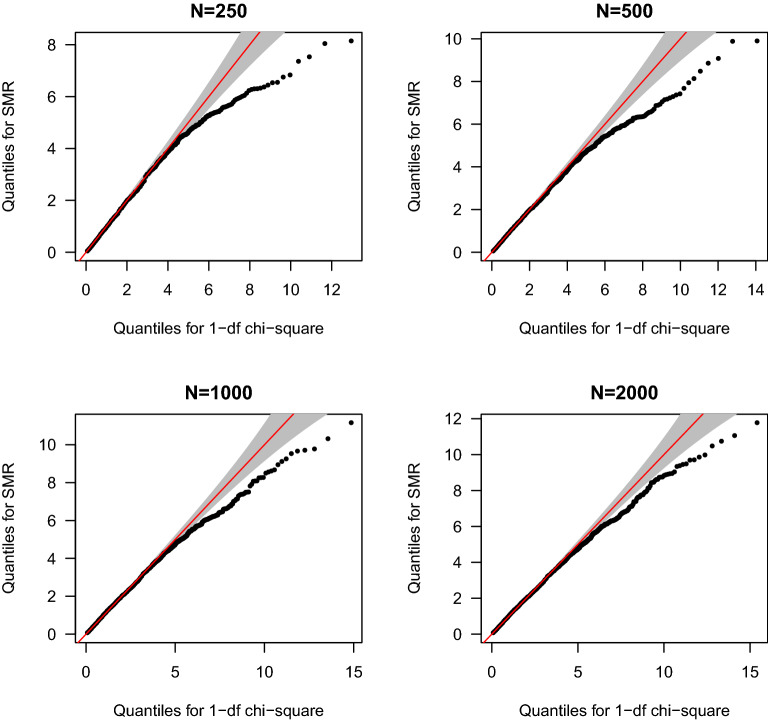
Quantile-quantile plot for simulated SMR statistics against statistics of 1-df chi-squire distribution. Instrumental eQTLs for SMR test were top associated eQTL ($p < 5 \times 10^{-8}$) selected from genes whose expression levels were simulated under a genetic model of 5 causal eQTLs and heritability of 0.4 at four different sample sizes (*N* = 250, 500, 1000, and 2000). The grey areas represent the 95% confidence band around 1-df chi-square statistics.

We performed extensive simulations to investigate the null distribution of the SMR statistic in a more realistic setting by using imputed GWAS genotype data from the Atherosclerosis Risk in Communities (ARIC) study of European-ancestry samples^9^. Specifically, we simulated gene expression levels for each Ensemble gene on autosomes at varying numbers of causal eQTLs (*n* = 1, 5, and 10), (narrow sense) heritability levels ($h^2$ = 0.1, 0.2, 0.4, 0.8), and sample sizes (*N* = 250, 500, 1000, and 2000). We tested association between all SNPs within each gene and expression levels of the gene, and only genes whose top associated SNP met the selection criteria ($p < 5 \times 10^{-8}$) were subjected to SMR test. GWAS association signals were randomly assigned from a standard normal distribution. Figure 3 shows the QQ plot for the SMR statistics when instrumental eQTLs were selected from genes with 5 causal eQTLs and a level of heritability = 0.4 at all four sample sizes. Clearly, the SMR statistics were lower than expected null values at the tail of distribution, though the distribution became closer to the null at larger sample size, which may be explained by the stronger eQTL signals as shown in our numerical example above. The complete set of QQ plots for the SMR test statistic are shown in Supplementary Figs. S1–S12 online. Overall, our simulations showed that the SMR statistics were conservative and did not strictly follow the 1-df chi-squire distribution, especially when the effect size of each individual eQTL was small on average. These results are consistent with our theoretical insights.

### More on SMR and a conditional test

One may want to use an estimate of $b_{gx}$ that takes into account the selection. However, such an estimate is not expected to be simple given the complexity of the selection (e.g., the SNP is the most significant one among a number of SNPs). Another alternative is to use another exposure GWAS independent of the exposure GWAS used in Step 1 to estimate $b_{gx}$ and then compute $T_\text {SMR}$. However, this is not recommended because $T_\text {SMR}$ is inherently conservative. $T_\text {SMR}$ is equal to the half of the harmonic mean of $W_{gx}$ and $W_{gy}$. Fixing one of $W_{gx}$ and $W_{gy}$, say $W_{gx}$, and change $W_{gy}$, $T_\text {SMR}$ reaches its smallest value $W_{gx}/2$ when $W_{gy} = W_{gx}$ and converges to $W_{gx}$ when $W_{gy}\rightarrow \infty$. The conservativeness of $T_\text {SMR}$ is also observed in simulation studies by Veturi and Ritchie^10^.

The null hypothesis for $T_\text {SMR}$ was not specifically defined in Zhu et al.^2^. It is unlikely to be the intended null $H_0: b_{xy}=0$. Actually, similar to the Sobel’s statistic popular in mediation analysis, the null corresponding to $T_\text {SMR}$ is $H_0: b_{gx}=0 \text { or } b_{gy}=0$. For this null, a statistic more powerful than $T_\text {SMR}$ is $\min \{W_{gx}, W_{gy}\}$. The statistic $\min \{W_{gx}, W_{gy}\}$ rejects the null $H_0: b_{gx}=0 \text { or } b_{gy}=0$ if and only if both $W_{gx}$ and $W_{gy}$ are significant. Therefore, whenever $\min \{W_{gx}, W_{gy}\}$ rejects the null, $T_\text {SMR}$ will but not vice versa. This is because $T_\text {SMR} < \min \{W_{gx}, W_{gy}\}$.

A test more powerful than $\min \{W_{gx}, W_{gy}\}$ (hence also more powerful than $T_\text {SMR}$) in the current situation is a conditional test. Because the SNP is selected for its significant association with the exposure, the situation $b_{gx}=0$ can be excluded. Given this information, a meaningful null would be $H_0: b_{gy}=0, b_{gx}\not =0$ for which a test statistic is $W_{gy}$. The null is rejected when $W_{gy}$ is significant. This test, conditional on a significant $W_{gx}$ statistic, assumes that there is no pleiotropy. That is, the selected SNP affects the trait only through the exposure and there are no other paths. In other words, the selected SNP is a valid instrument. In light of Eq. (1), $b_{gy}=0$ if and only if $b_{xy}=0$ when the possibility of $b_{gx}=0$ is excluded. Hence the null for this conditional test is equivalent to $H_0: b_{xy}=0$. This test is asymptotically valid because $W_{gy}$ asymptotically follows a 1-df chi-square distribution. The threshold for significance for this test is not at the genome level. Rather, it is at the gene level and only needs to be corrected for the number of genes for which SNPs are selected for instruments. This results in a more powerful testing procedure than using a genome-wide threshold.

### An empirical study

We compared the performance of conditional test we proposed and the SMR test on an empirical study of schizophrenia. We used to-date the largest GWAS summary statistics for schizophrenia^11^ and the eQTL results from analysis of 1387 brain samples (prefrontal cortex) by the PsychENCODE^12^ (downloaded from the SMR data resource website). In total, 9639 genes were tested for SMR at a top associated *cis*-eQTL ($p < 5 \times 10^{-8}$) and 65 genes were significant after Bonferroni correction. In contrast, the conditional test, whose test statistic is $W_{gy}$ and considers only those instrumental eQTLs, discovered 127 Bonferroni-significant genes, including 62 genes not detected by SMR ($p<0.05/9639 = 5.18726\times 10^{-6}$. Supplementary Table S1 online). Among those genes missed by SMR, there were several strong candidates for schizophrenia, such as *AKT3*^13–15^, *RGS6*^16,17^, and *KCNN3*. It may not be surprising that *AKT3* and *RGS6* were identified as these two harbored genome-wide significant variants ($p < 5 \times 10^{-8}$) in original GWAS^11^, but the discovery of *KCNN3* was novel and the strongest SNP-level association evidence for this gene was only at $p = 9 \times 10^{-7}$ (rs10796933). Of note, our previous study also showed evidence for the association of *KCNN3* with schizophrenia through integrated analysis of GWAS with methylation QTL^18^.

### Two-sample MR Steiger method

The two-sample MR Steiger method^1,19^ assumes that there is a causal relationship between the exposure and the trait and that the selected SNP is a valid instrument for one of them (but it is unknown for which one)^1^. A SNP is selected not only for its association with the exposure but also for its association with the trait^1,19^. The null for the two-sample MR Steiger test is $H_0: \rho _{gx} = \rho _{gy}$ where $\rho _{gx}=Corr(g, x)$ and $\rho _{gy}=Corr(g, y)$ are the (population) Pearson correlation coefficients. Let $\hat{\rho }_{gx}$ and $\hat{\rho }_{gy}$ be the sample correlation coefficients corresponding to $\rho _{gx}$ and $\rho _{gy}$, respectively. Using Fisher’s Z transformation, there are

2 $$\begin{aligned} z_{gx}&:= \frac{1}{2}\ln \frac{1+|\hat{\rho }_{gx}|}{1-|\hat{\rho }_{gx}|} \nonumber \\&\quad \sim N\left( \frac{1}{2}\ln \frac{1+|\rho _{gx}|}{1-|\rho _{gx}|}, \frac{1}{n_x-3}\right) , \text { and} \end{aligned}$$

3 $$\begin{aligned} z_{gy}&:= \frac{1}{2}\ln \frac{1+|\hat{\rho }_{gy}|}{1-|\hat{\rho }_{gy}|} \nonumber \\&\quad \sim N\left( \frac{1}{2}\ln \frac{1+|\rho _{gy}|}{1-|\rho _{gy}|}, \frac{1}{n_y-3}\right) , \end{aligned}$$

where $n_x$ and $n_y$ are sample sizes. The null $H_0: \rho _{gx} = \rho _{gy}$ is equivalent to saying that the mean of $z_{qx}$ is equal to the mean of $z_{qy}$. The two-sample MR Steiger method uses the following statistic^1,19^:

$$\begin{aligned} T_\text {Steiger} = \frac{z_{gx} - z_{gy}}{\sqrt{1/(n_x-3) + 1/(n_y-3)}} \sim N(0,1). \end{aligned}$$

If $T_\text {Steiger}$ is significant and positive, the causal direction is from *x* to *y*. If $T_\text {Steiger}$ is significant and negative, the causal direction is from *y* to *x*.

However, the statistic $T_\text {Steiger}$ does not approximately follow a standard normal distribution because the SNP is selected for its significant *p*-values. Using a selection criterion $p<5\times 10^{-8}$, or $W_{gx}$ and $W_{gy}$ greater than 29.71679 on 1-df chi-square scale, the sample correlation coefficients $|\hat{\rho }_{gx}|$ and $|\hat{\rho }_{gy}|$ would be at least 0.4823663, 0.1700451, or 0.05443772 for $n_x=$ 100, 1000, or 10,000 given the relationship $|\hat{\rho }_{gx}| = 1/\sqrt{1+(n_x-2)/W_{gx}}$. Although this selection procedure is useful for selecting the instrument SNP, it imposes a lower limit on $|\hat{\rho }_{gx}|$ and $|\hat{\rho }_{gy}|$. $|\hat{\rho }_{gx}| \left( |\hat{\rho }_{gy}|\right)$ over-estimates $|\rho _{gx}| \left( |\rho _{gy}|\right)$ and is not consistent. The mean of the statistic $T_\text {Steiger}$ is not around 0 even when $H_0: \rho _{gx} = \rho _{gy}$ holds if $n_x\not =n_y$. The distribution of $z_{gx}$ is truncated and is not normal. So is the distribution of $z_{gy}$. The variance of $z_{gx}$ is smaller than $1/(n_x-3)$ due to selection. Similarly, the variance of $z_{gy}$ is smaller than $1/(n_y-3)$. When $n_x=n_y$, the numerator of $T_\text {Steiger}$ is around 0 and $T_\text {Steiger}$ is conservative. When $n_x\not =n_y$, the numerator of $T_\text {Steiger}$ is no longer around 0 and $T_\text {Steiger}$ is liberal. Overall, the distributions of $z_{gx}$ and $z_{gy}$ are truncated normal instead of normal. The argument that the statistic $T_\text {Steiger}$ follows asymptotically a standard normal does not hold. The two-sample MR Steiger method can be either liberal or conservative.

**Figure 4:**
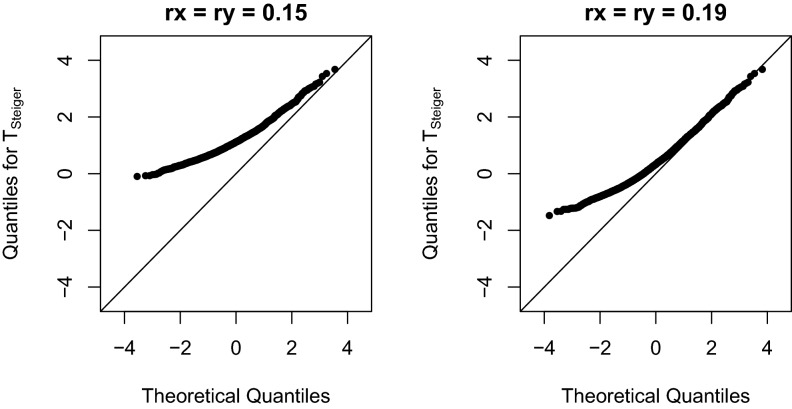
Normal Q-Q plot of simulated $T_\text {Steiger}$ with $n_x=1000, n_y=10{,}000$. $\{(z_{gx}, z_{gy})\}$ are selected from 10,000 replicates at genome-wide significance level $5\times 10^{-8}$.

Numerical examples are constructed. First we consider the case $n_x = 1000, n_y = 10{,}000$ and demonstrate the effect of selection severity. Ten thousand random samples of $z_{gx}$ and $z_{gy}$ are independently generated from the normal distributions shown in Eqs. (2) and (3). These $z_{gx}$ and $z_{gy}$ form a $10{,}000\times 2$ matrix. The first column contains values for $z_{gx}$ and the second for $z_{gy}$. Only the rows satisfying $z_{gx}\ge 0.5\ln [(1+0.1700451)/(1-0.1700451)]=0.17171315$ and $z_{gy}\ge 0.5\ln [(1+0.05443772)/(1-0.05443772)]=0.05449159$ are kept. This selection criterion corresponds to $5\times 10^{-8}$ on the *p*-value scale. When $\rho _{gx}=\rho _{gy}=0.15$, there are 2557 $(z_{gx}, z_{gy})$ selected on which the statistic $T_\text {Steiger}$ is computed. The sample mean of selected $\{z_{gx}\}$ is 0.1903508 while the sample mean of selected $\{z_{gy}\}$ ($=0.1512602$) is lower, as expected. A normal quantile-quantile plot of $T_\text {Steiger}$ is shown in the left panel of Fig. 4. Clearly the distribution of $T_\text {Steiger}$ is different from normal. Type I error rates are inflated. At significance level 0.05 and 0.01, the type I error rates (i.e., the proportion of significant $T_\text {Steiger}$ statistics) are 0.08916699 and 0.01486117, respectively. If $\rho _{gx}=\rho _{gy}=0.19$, the selection is less severe. Almost 75% (7434 out of 10,000) $(z_{gx}, z_{gy})$s are selected. Even so, the distribution of $T_\text {Steiger}$ shows apparent departure from normal as shown in the right panel of Fig. 4. At significance level 0.05 and 0.01, the type I error rates are 0.0306699 and 0.005111649, respectively. In this case, $T_\text {Steiger}$ appears to be conservative.

**Figure 5:**
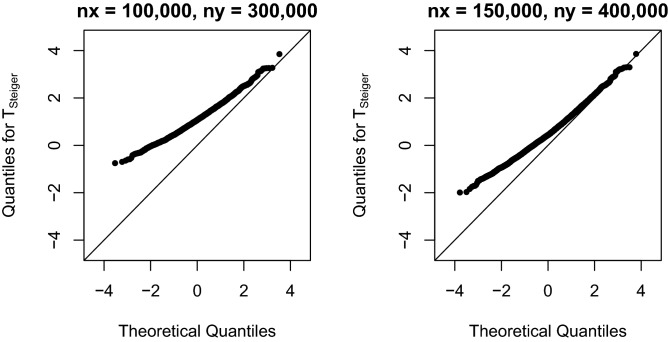
Normal Q-Q plot of simulated $T_\text {Steiger}$ with $\rho _{gx}=\rho _{gy}=0.015$. $\{(z_{gx}, z_{gy})\}$ are selected from 10,000 replicates at genome-wide significance level $5\times 10^{-8}$.

We also considered larger sample sizes. When $n_x=100{,}000, n_y=300{,}000$, and $\rho _{gx}=\rho _{gy}=0.015$, 2,375 $(z_{gx}, z_{gy})$s are selected. When $n_x=150{,}000, n_y=400{,}000$ and $\rho _{gx}=\rho _{gy}=0.015$, 6,410 $(z_{gx}, z_{gy})$s are selected. As shown in Fig. 5, there is apparent departure of the distribution of $T_\text {Steiger}$ from a normal. At significance level 0.05, the type I error rate is 0.09515789 for the case $n_x=100{,}000, n_y=300{,}000$ and is 0.03728549 for $n_x=100,100, n_y=400{,}000$. At significance level 0.01, the type I error rates are 0.01515789 and 0.006084243, respectively. The type I error rates can be either inflated or deflated.

One remedy would be to estimate $\rho _{gx}$ and $\rho _{gy}$ by maximizing the conditional likelihood given the SNP selection criteria. Let $\phi (\cdot )$ and $\Phi (\cdot )$ denote the density function and the distribution function of the standard normal, respectively. The likelihood ratio statistic for testing $H_0$ is $2\log (L_1/L_0)$ where

$$\begin{aligned} L_1&= \left[ \max _{\mu _{gx}}\frac{\phi (\sqrt{n_x-3}(z_{gx} - \mu _{gx}))}{1-\Phi (\sqrt{n_x-3}(c_{gx} - \mu _{gx}))}\right] \cdot \left[ \max _{\mu _{gy}}\frac{\phi (\sqrt{n_y-3}(z_{gy} - \mu _{gy}))}{1-\Phi (\sqrt{n_y-3}(c_{gy} - \mu _{gy}))}\right] , \\ L_0&= \max _{\mu _g}\left[ \frac{\phi (\sqrt{n_x-3}(z_{gx} - \mu _g))}{1-\Phi (\sqrt{n_x-3}(c_{gx} - \mu _g))} \cdot \frac{\phi (\sqrt{n_y-3}(z_{gy} - \mu _g))}{1-\Phi (\sqrt{n_y-3}(c_{gy} - \mu _g))}\right] \end{aligned}$$

with $c_{gx}$ and $c_{gy}$ selection thresholds corresponding to $z_{gx}$ and $z_{gy}$, respectively. However, due to selection, computation of $L_1$ and $L_0$ can be challenging. One alternative method is to use an exposure GWAS and a trait GWAS that are independent of those used to select the SNP. However, such studies may be impractical to obtain^6^.

## Discussion

Summary statistics MR is subject to selection bias, resulting in excessive false positives (for instance, the MR Steiger method) or missed discoveries (for instance, the SMR method). This bias is a form of winner’s curse. Selection bias has been discussed in the literature in the context of the choice of the instrument SNPs^7^, colocalisation test^20^, and estimation of exposure effect^5,6^.

Our work complements previous studies on selection bias due to selection of SNPs. While previous work focused on the effect of this bias on the Wald ratio^5,6^ (i.e., estimation), ours focuses on testing whether the exposure causally affects the outcome (i.e., inference). Selection bias leads to underestimation of the Wald ratio^5^ but its effect on type I error rate can be either liberal or conservative depending on the MR method used. Most importantly, the SMR method is conservative even in the absence of selection bias where ${\hat{b}}_{gx}$ is approximately normal.

Correcting for selection bias is a challenging task. Zhao et al.^6^ get around this issue by using an independent exposure GWAS. On the other hand, our conditional test, an alternative to the SMR method, uses only the trait GWAS. It may be expanded to accommodate multiple instrumental SNPs and the presence of pleiotropy.

## Supplementary Information

Supplementary Information.
