# Supplementary material for: Effect of selection bias on two sample summary data based Mendelian randomization
Source: Sci Rep. 2021 Apr 7;11:7585. doi: 10.1038/s41598-021-87219-6 (PMC8027662; doi:10.1038/s41598-021-87219-6)
Supplement: Supplementary file 1 — Supplementary Information. [file 41598_2021_87219_MOESM1_ESM.docx]

Supplementary Information

**Effect of selection bias on two sample summary data based Mendelian randomization
Kai Wang**1,* **and Shizhong Han**2,3

1Department of Biostatistics, The University of Iowa, Iowa City, 52242, US
2Lieber Institute for Brain Development, Johns Hopkins School of Medicine, Baltimore, 21205, US 3Department of Psychiatry and Behavioral Sciences, Johns Hopkins School of Medicine, Baltimore, 21205, US

* kai-wang@uiowa.edu


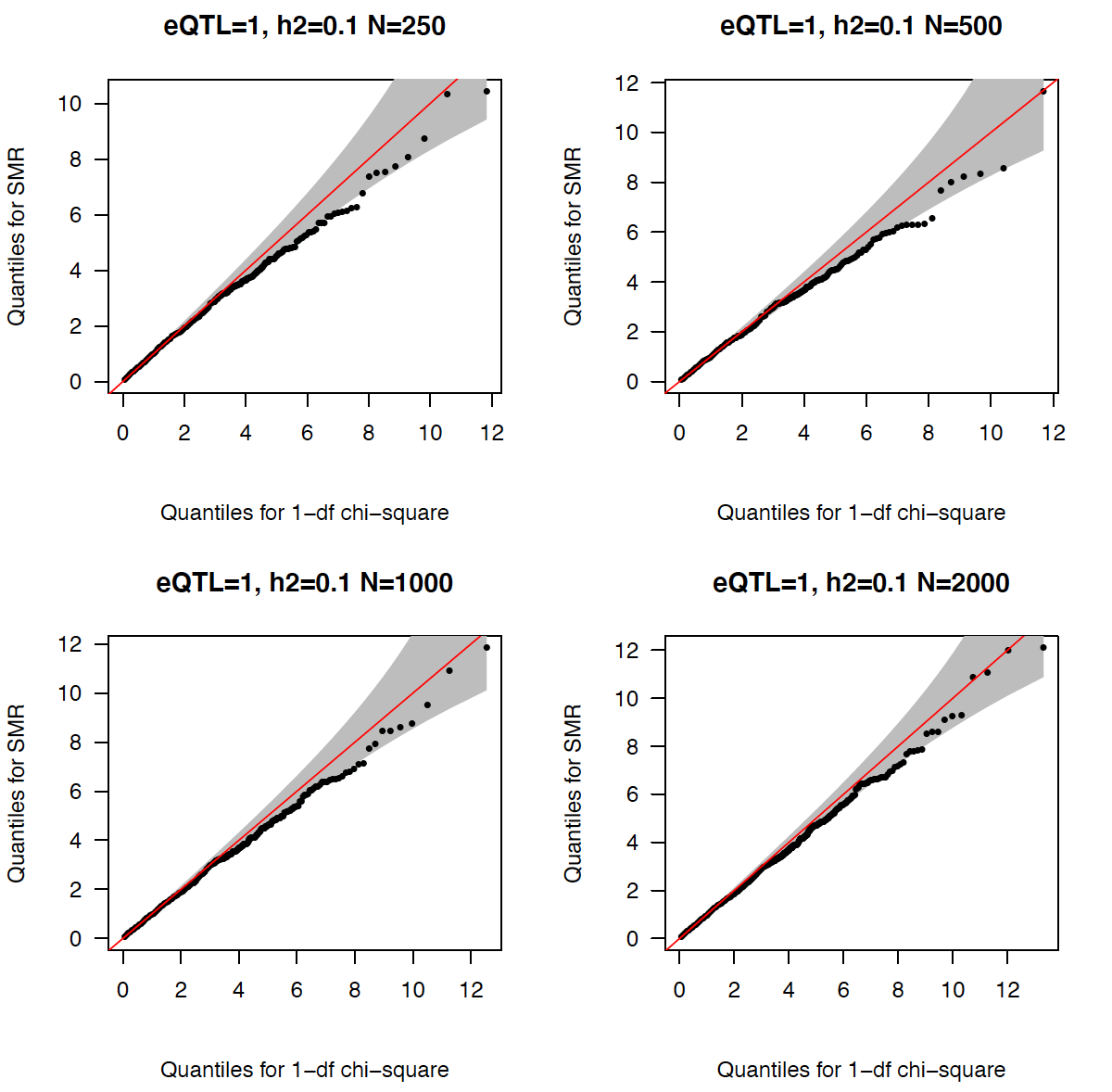


Supplementary Figure S1: Quantile-quantile plot for simulated SMR statistics against statistics of 1-df chi-squire distribution. Instrumental eQTLs for SMR test were top associated eQTL ($\boldsymbol{p<5\times}\boldsymbol{10}^{\boldsymbol{-8}}$) selected from genes whose expression levels were simulated under a genetic model in which the number of causal eQTL is 1 (eQTL =1) and the heritability is 0.1 (h2 = 0.1) at four different sample sizes (N = 250, 500, 1000, and 2000). The grey areas represent the 95% confidence band around 1-df chi-square statistics.


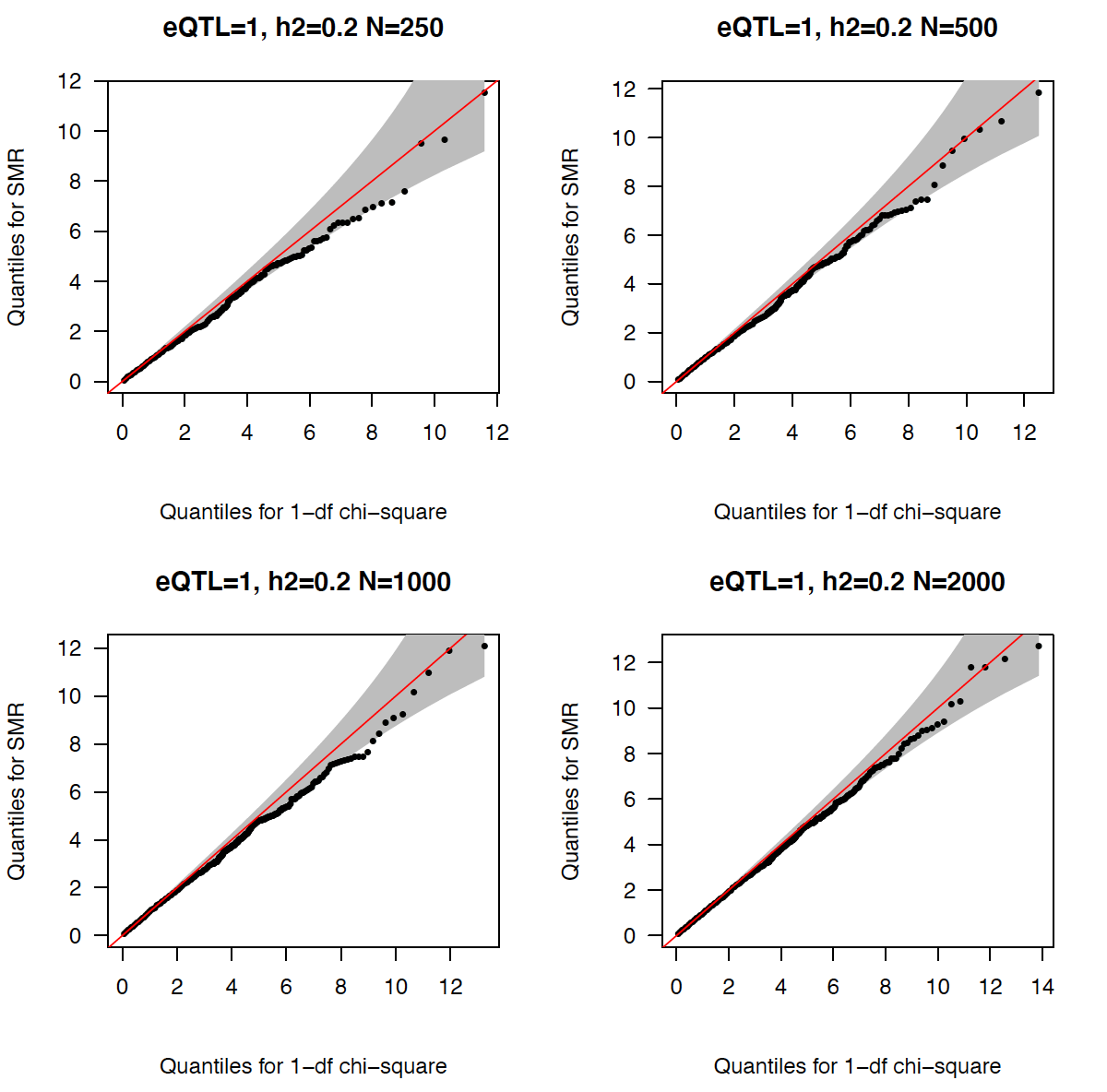


Supplementary Figure S2: Quantile-quantile plot for simulated SMR statistics against statistics of 1-df chi-squire distribution. Instrumental eQTLs for SMR test were top associated eQTL ($\boldsymbol{p<5\times}\boldsymbol{10}^{\boldsymbol{-8}}$) selected from genes whose expression levels were simulated under a genetic model in which the number of causal eQTL is 1 (eQTL =1) and the heritability is 0.2 (h2 = 0.2) at four different sample sizes (N = 250, 500, 1000, and 2000). The grey areas represent the 95% confidence band around 1-df chi-square statistics.


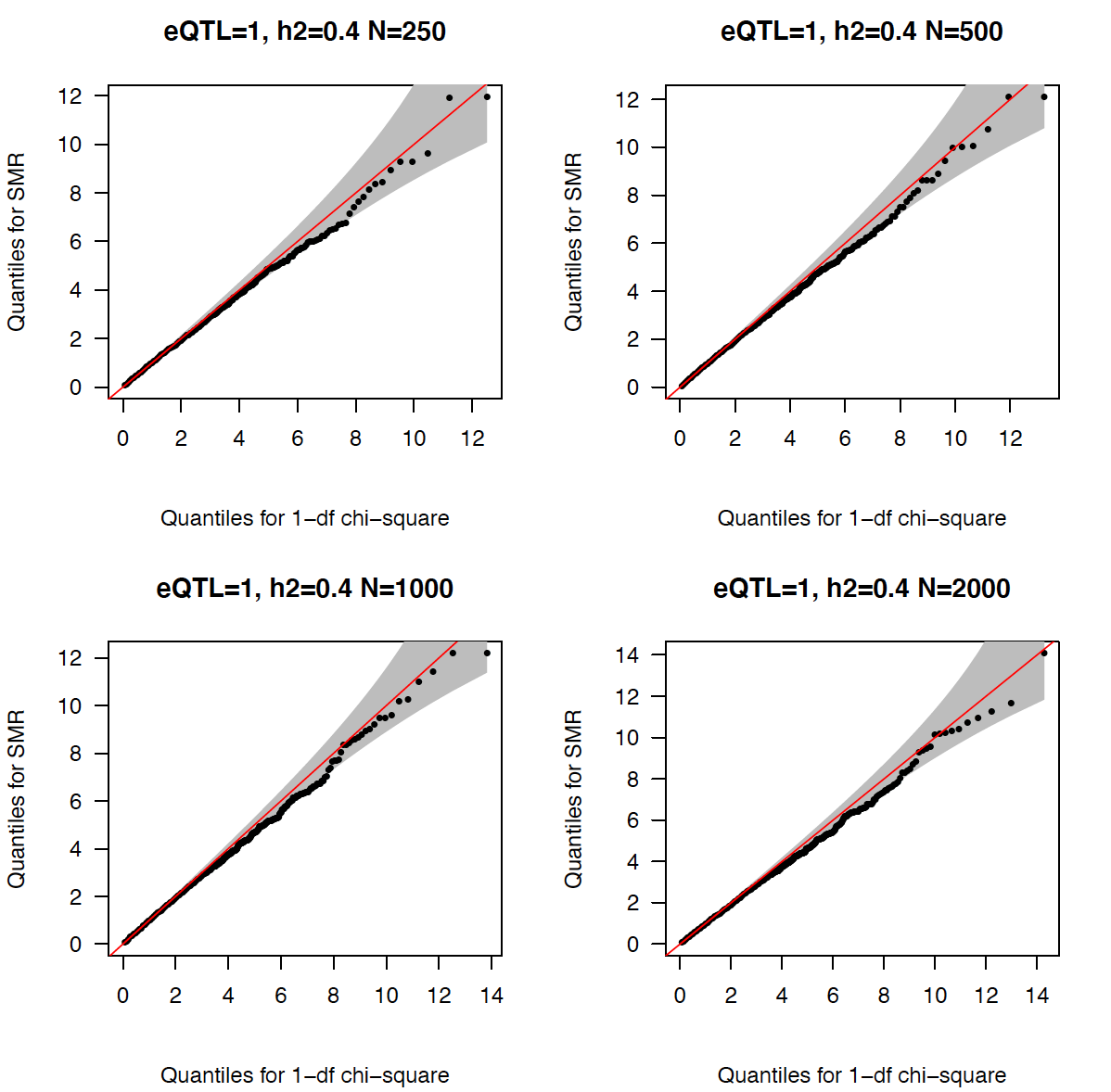


Supplementary Figure S3: Quantile-quantile plot for simulated SMR statistics against statistics of 1-df chi-squire distribution. Instrumental eQTLs for SMR test were top associated eQTL ($\boldsymbol{p<5\times}\boldsymbol{10}^{\boldsymbol{-8}}$) selected from genes whose expression levels were simulated under a genetic model in which the number of causal eQTL is 1 (eQTL =1) and the heritability is 0.4 (h2 = 0.4) at four different sample sizes (N = 250, 500, 1000, and 2000). The grey areas represent the 95% confidence band around 1-df chi-square statistics.


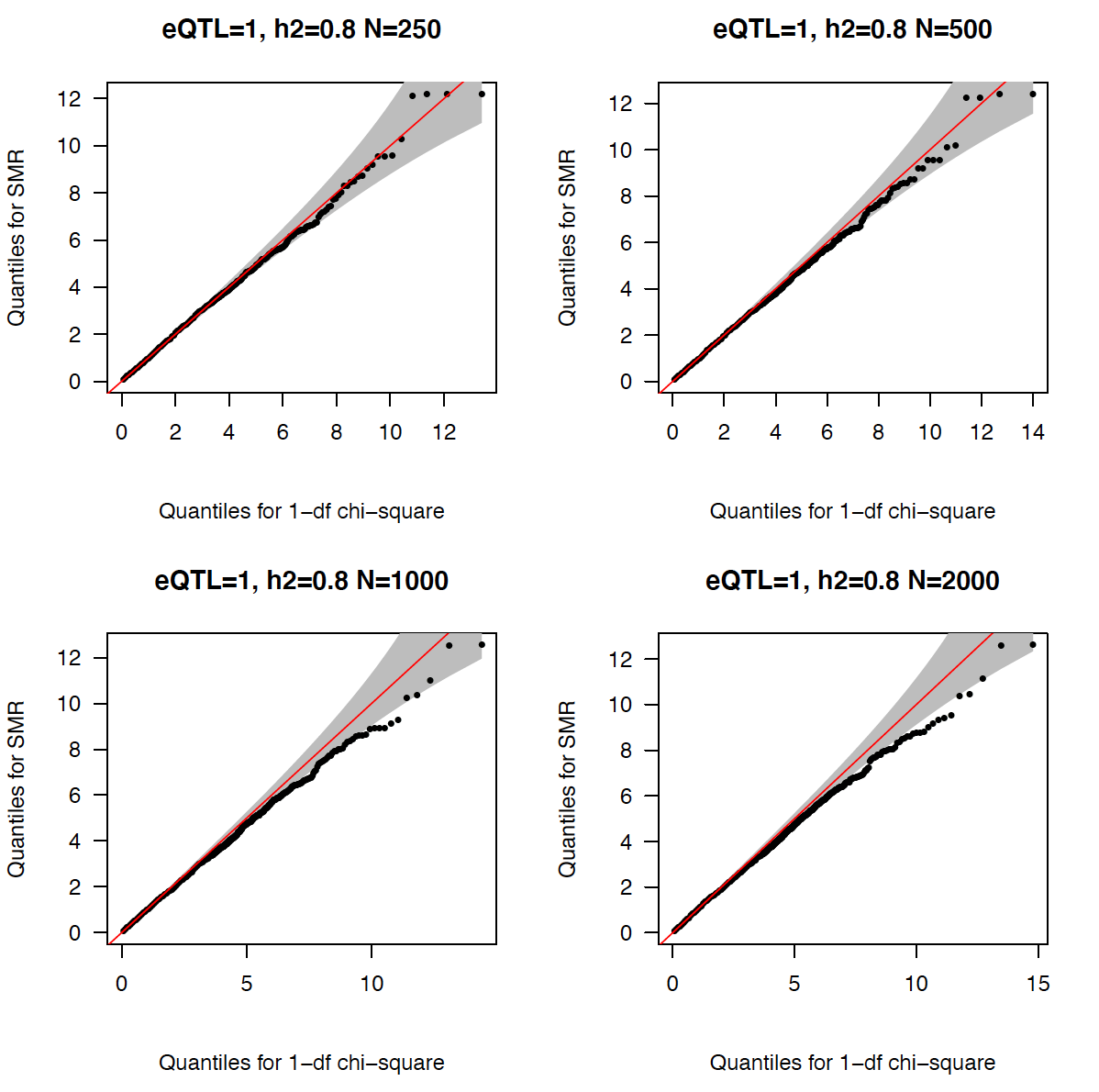


Supplementary Figure S4: Quantile-quantile plot for simulated SMR statistics against statistics of 1-df chi-squire distribution. Instrumental eQTLs for SMR test were top associated eQTL ($\boldsymbol{p<5\times}\boldsymbol{10}^{\boldsymbol{-8}}$) selected from genes whose expression levels were simulated under a genetic model in which the number of causal eQTL is 1 (eQTL =1) and the heritability is 0.8 (h2 = 0.8) at four different sample sizes (N = 250, 500, 1000, and 2000). The grey areas represent the 95% confidence band around 1-df chi-square statistics.


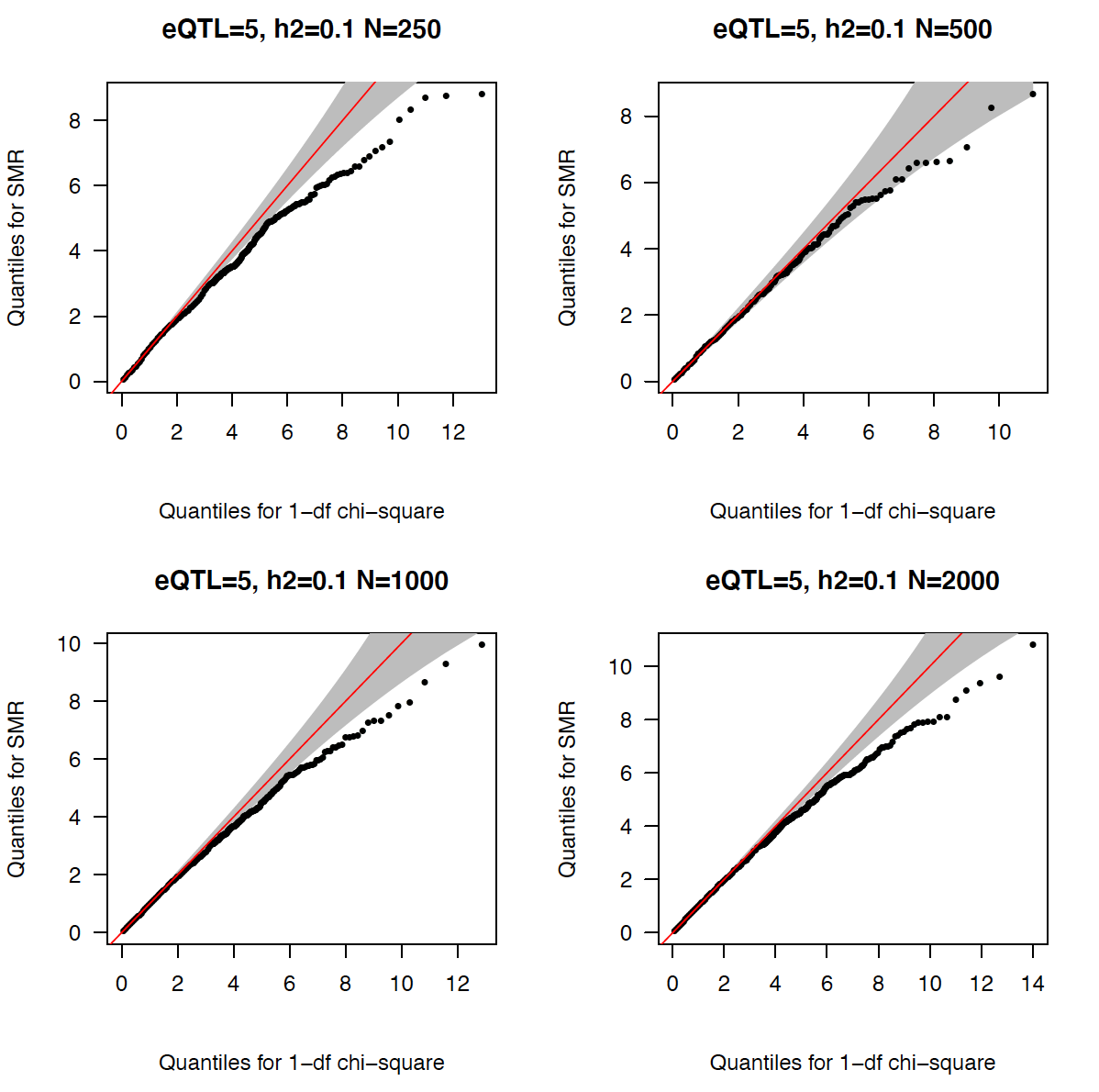


Supplementary Figure S5: Quantile-quantile plot for simulated SMR statistics against statistics of 1-df chi-squire distribution. Instrumental eQTLs for SMR test were top associated eQTL ($\boldsymbol{p<5\times}\boldsymbol{10}^{\boldsymbol{-8}}$) selected from genes whose expression levels were simulated under a genetic model in which the number of causal eQTLs is 5 (eQTL =5) and the heritability is 0.1 (h2 = 0.1) at four different sample sizes (N = 250, 500, 1000, and 2000). The grey areas represent the 95% confidence band around 1-df chi-square statistics.


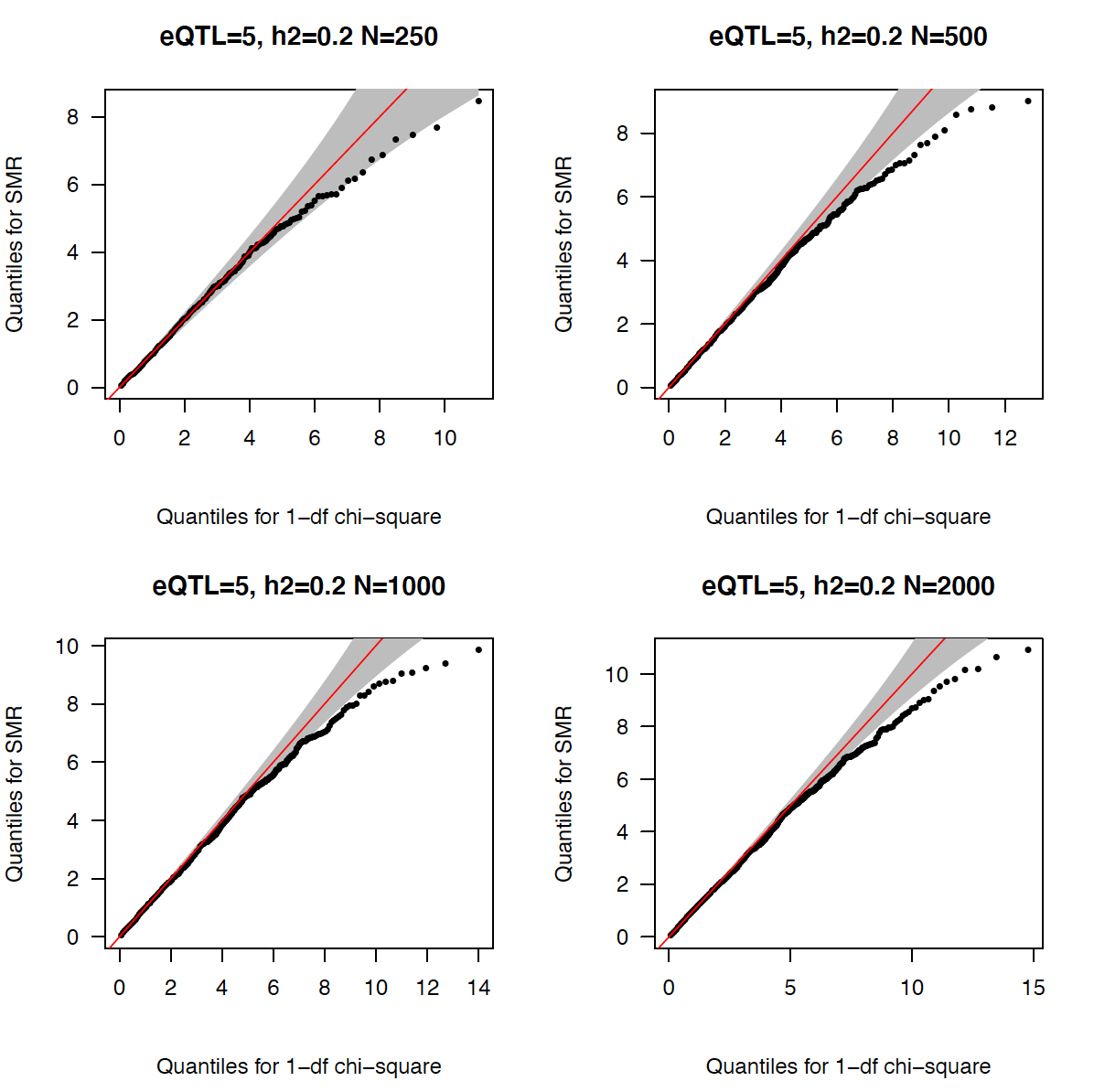


Supplementary Figure S6: Quantile-quantile plot for simulated SMR statistics against statistics of 1-df chi-squire distribution. Instrumental eQTLs for SMR test were top associated eQTL ($\boldsymbol{p<5\times}\boldsymbol{10}^{\boldsymbol{-8}}$) selected from genes whose expression levels were simulated under a genetic model in which the number of causal eQTLs is 5 (eQTL =5) and the heritability is 0.2 (h2 = 0.2) at four different sample sizes (N = 250, 500, 1000, and 2000). The grey areas represent the 95% confidence band around 1-df chi-square statistics.


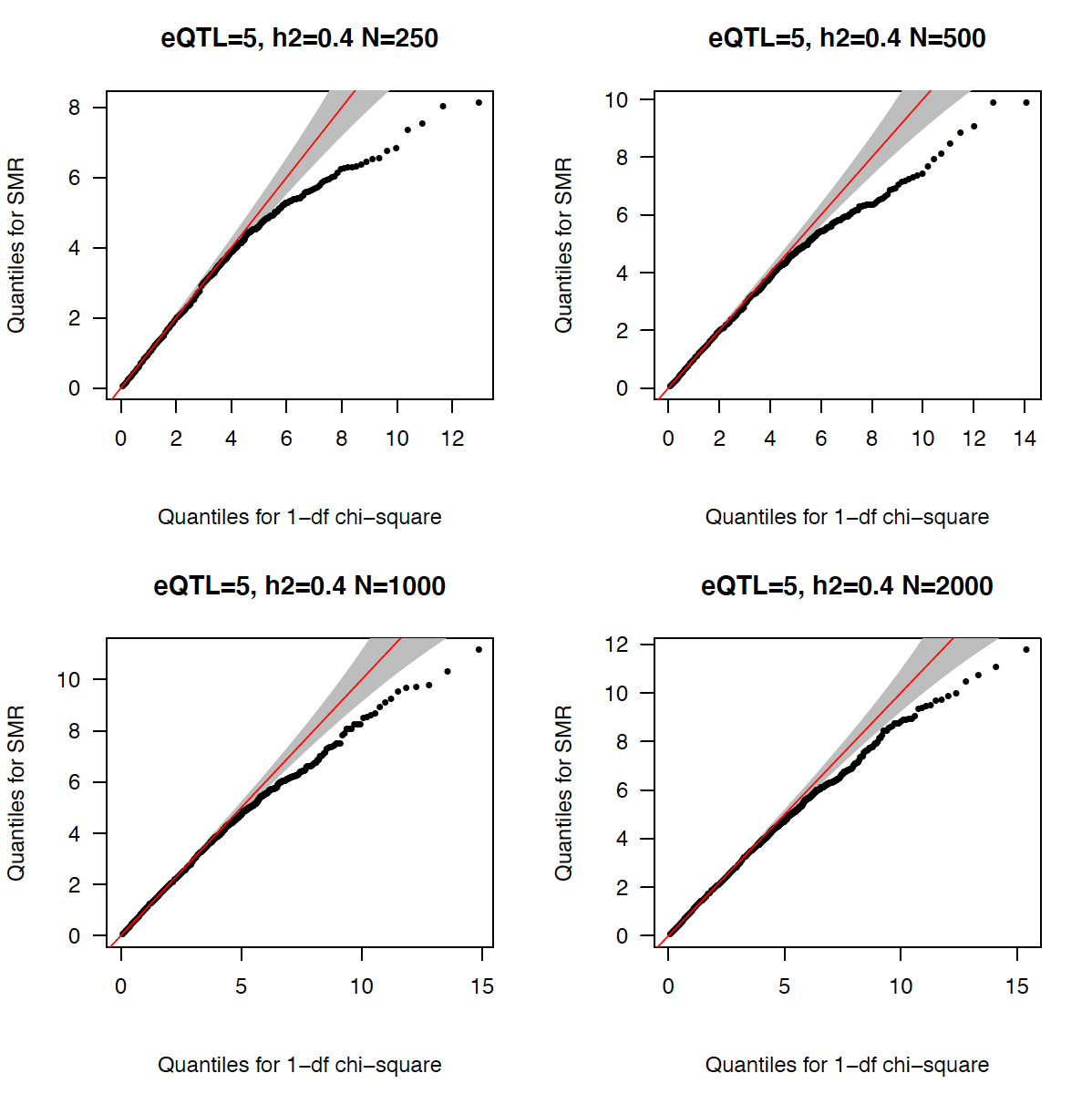


Supplementary Figure S7: Quantile-quantile plot for simulated SMR statistics against statistics of 1-df chi-squire distribution. Instrumental eQTLs for SMR test were top associated eQTL ($\boldsymbol{p<5\times}\boldsymbol{10}^{\boldsymbol{-8}}$) selected from genes whose expression levels were simulated under a genetic model in which the number of causal eQTLs is 5 (eQTL =5) and the heritability is 0.4 (h2 = 0.4) at four different sample sizes (N = 250, 500, 1000, and 2000). The grey areas represent the 95% confidence band around 1-df chi-square statistics.


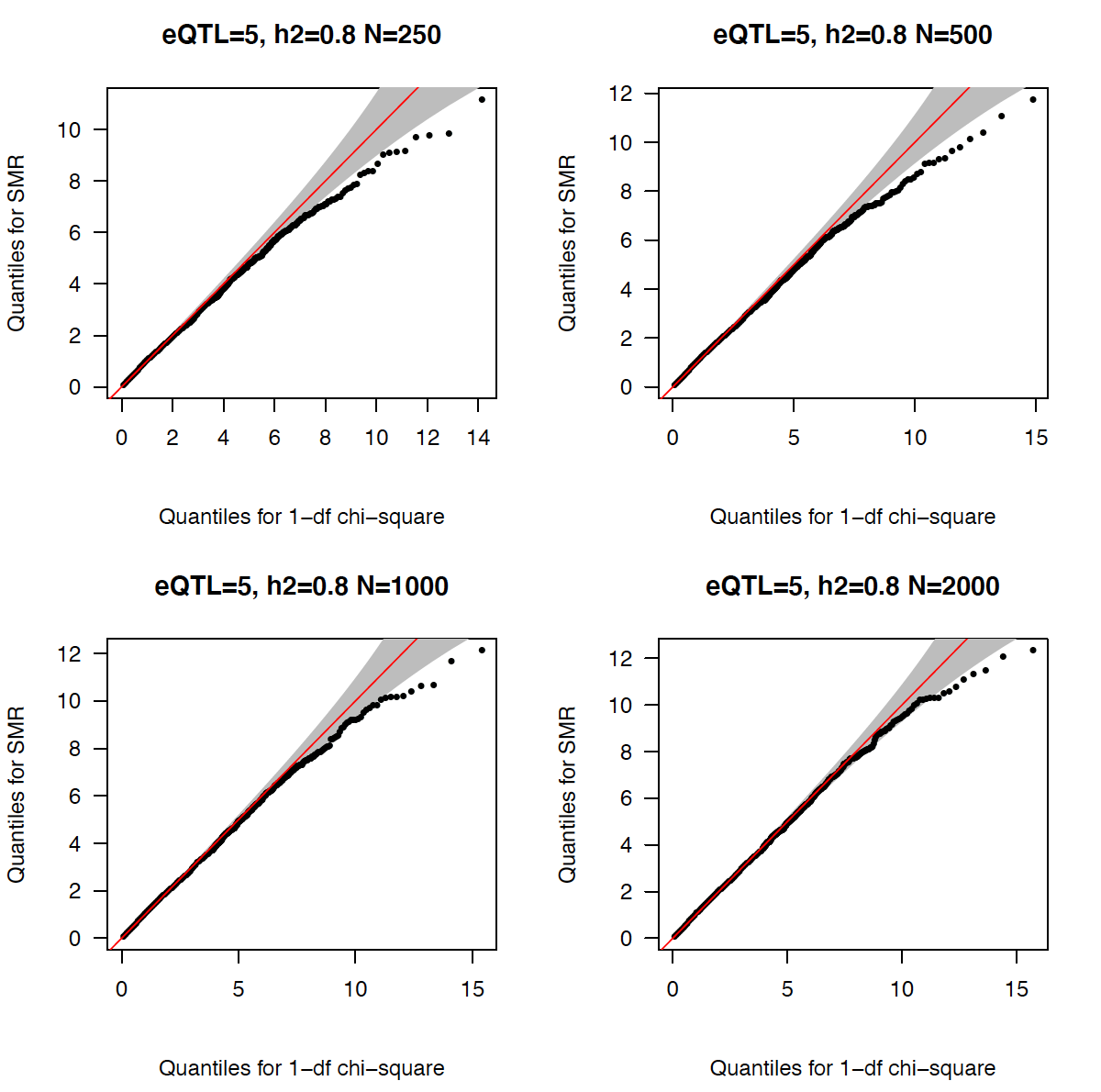


Supplementary Figure S8: Quantile-quantile plot for simulated SMR statistics against statistics of 1-df chi-squire distribution. Instrumental eQTLs for SMR test were top associated eQTL ($\boldsymbol{p<5\times}\boldsymbol{10}^{\boldsymbol{-8}}$) selected from genes whose expression levels were simulated under a genetic model in which the number of causal eQTLs is 5 (eQTL =5) and the heritability is 0.8 (h2 = 0.8) at four different sample sizes (N = 250, 500, 1000, and 2000). The grey areas represent the 95% confidence band around 1-df chi-square statistics.


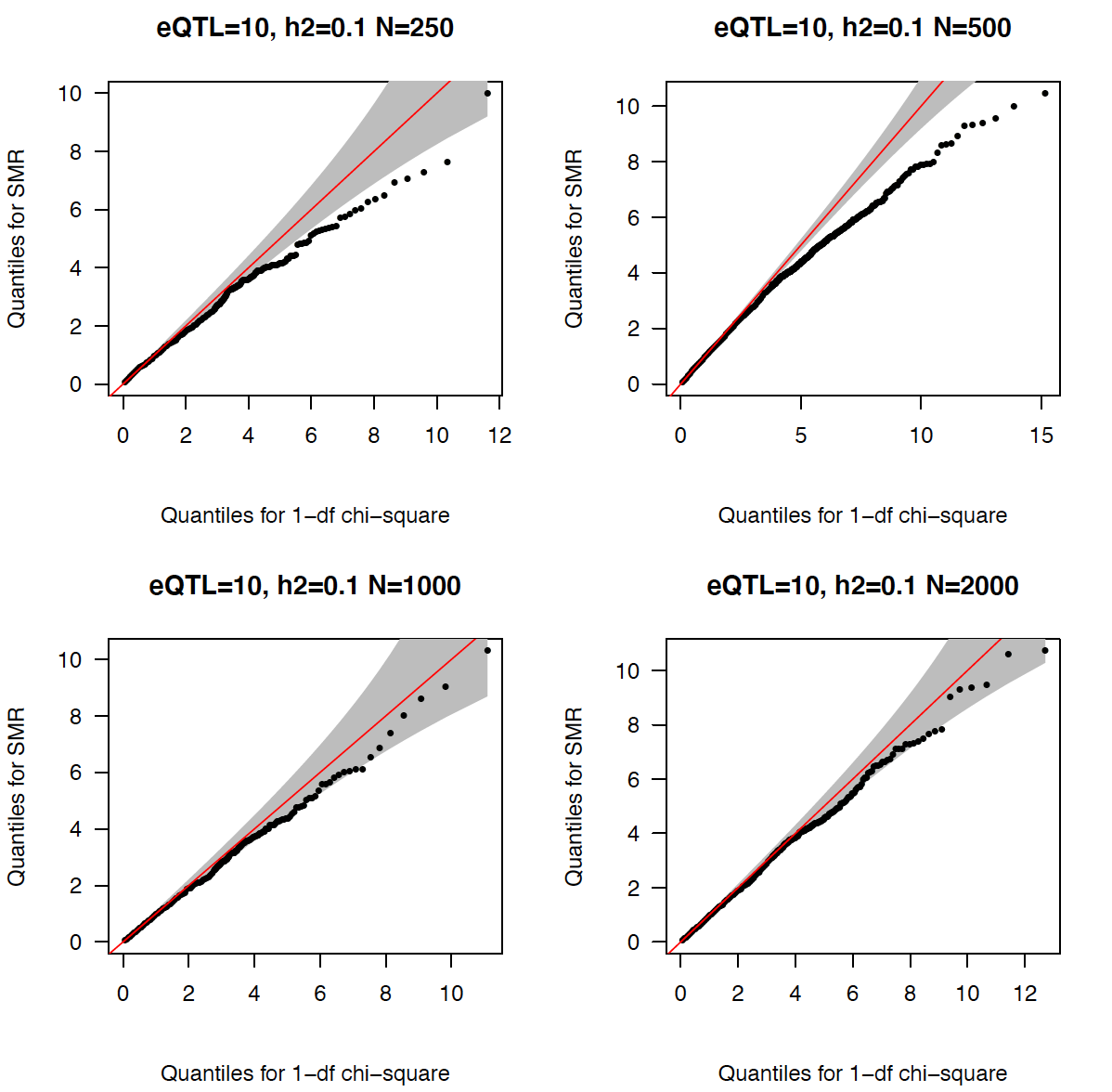


Supplementary Figure S9: Quantile-quantile plot for simulated SMR statistics against statistics of 1-df chi-squire distribution. Instrumental eQTLs for SMR test were top associated eQTL ($\boldsymbol{p<5\times}\boldsymbol{10}^{\boldsymbol{-8}}$) selected from genes whose expression levels were simulated under a genetic model in which the number of causal eQTLs is 10 (eQTL =10) and the heritability is 0.1 (h2 = 0.1) at four different sample sizes (N = 250, 500, 1000, and 2000). The grey areas represent the 95% confidence band around 1-df chi-square statistics.


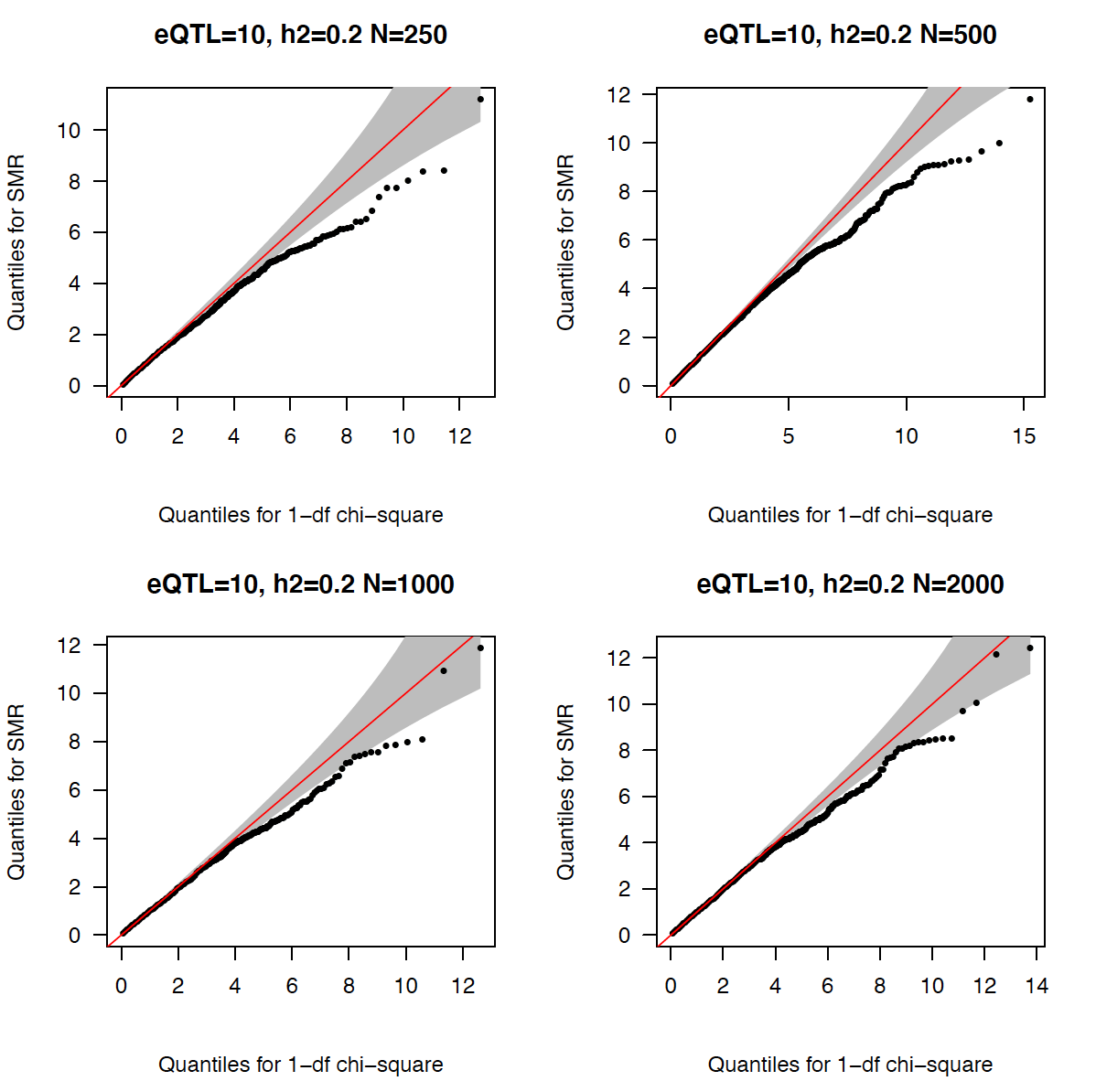


Supplementary Figure S10: Quantile-quantile plot for simulated SMR statistics against statistics of 1-df chi-squire distribution. Instrumental eQTLs for SMR test were top associated eQTL ($\boldsymbol{p<5\times}\boldsymbol{10}^{\boldsymbol{-8}}$) selected from genes whose expression levels were simulated under a genetic model in which the number of causal eQTLs is 10 (eQTL =10) and the heritability is 0.2 (h2 = 0.2) at four different sample sizes (N = 250, 500, 1000, and 2000). The grey areas represent the 95% confidence band around 1-df chi-square statistics.


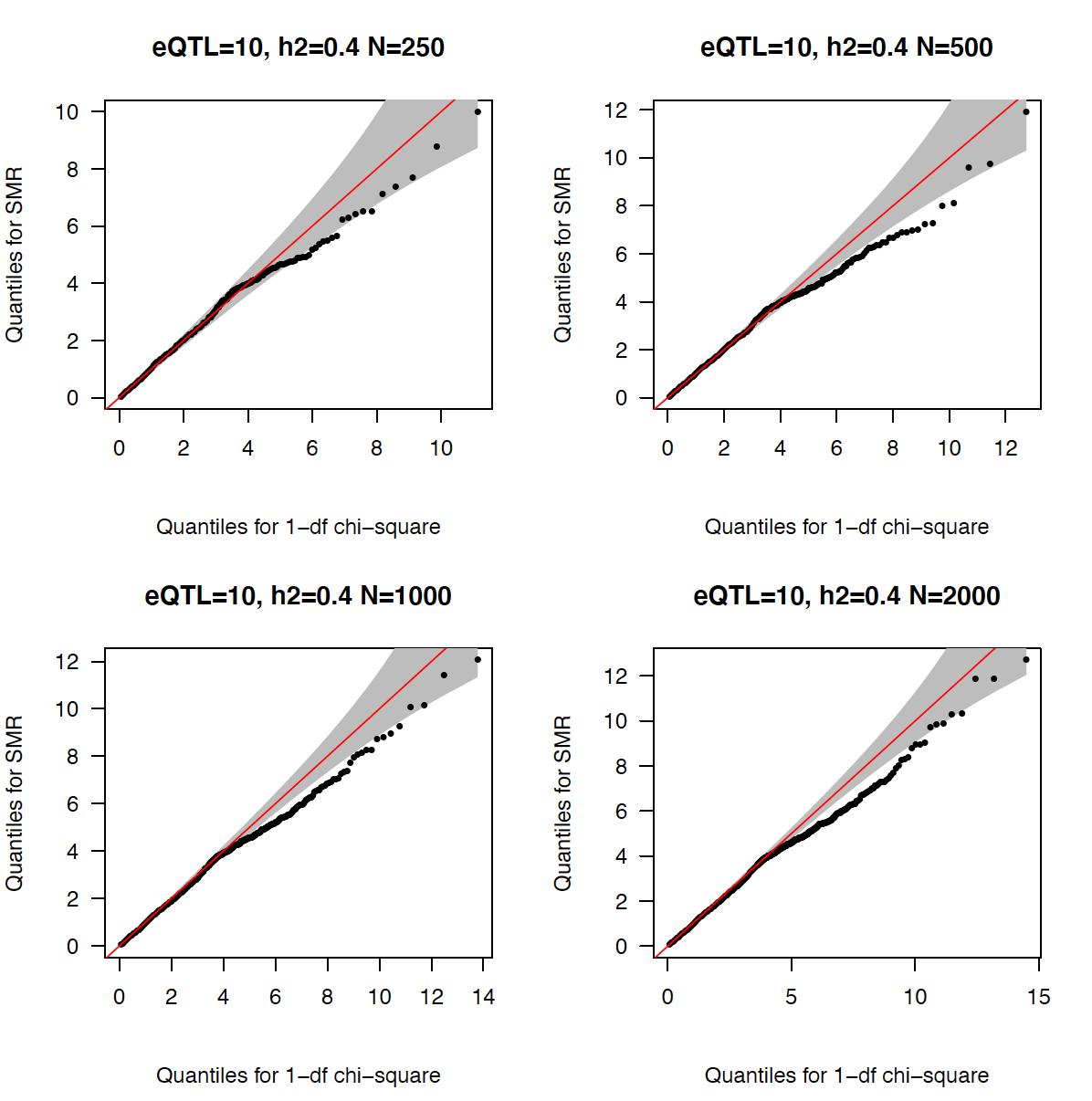


Supplementary Figure S11: Quantile-quantile plot for simulated SMR statistics against statistics of 1-df chi-squire distribution. Instrumental eQTLs for SMR test were top associated eQTL ($\boldsymbol{p<5\times}\boldsymbol{10}^{\boldsymbol{-8}}$) selected from genes whose expression levels were simulated under a genetic model in which the number of causal eQTLs is 10 (eQTL =10) and the heritability is 0.4 (h2 = 0.4) at four different sample sizes (N = 250, 500, 1000, and 2000). The grey areas represent the 95% confidence band around 1-df chi-square statistics.


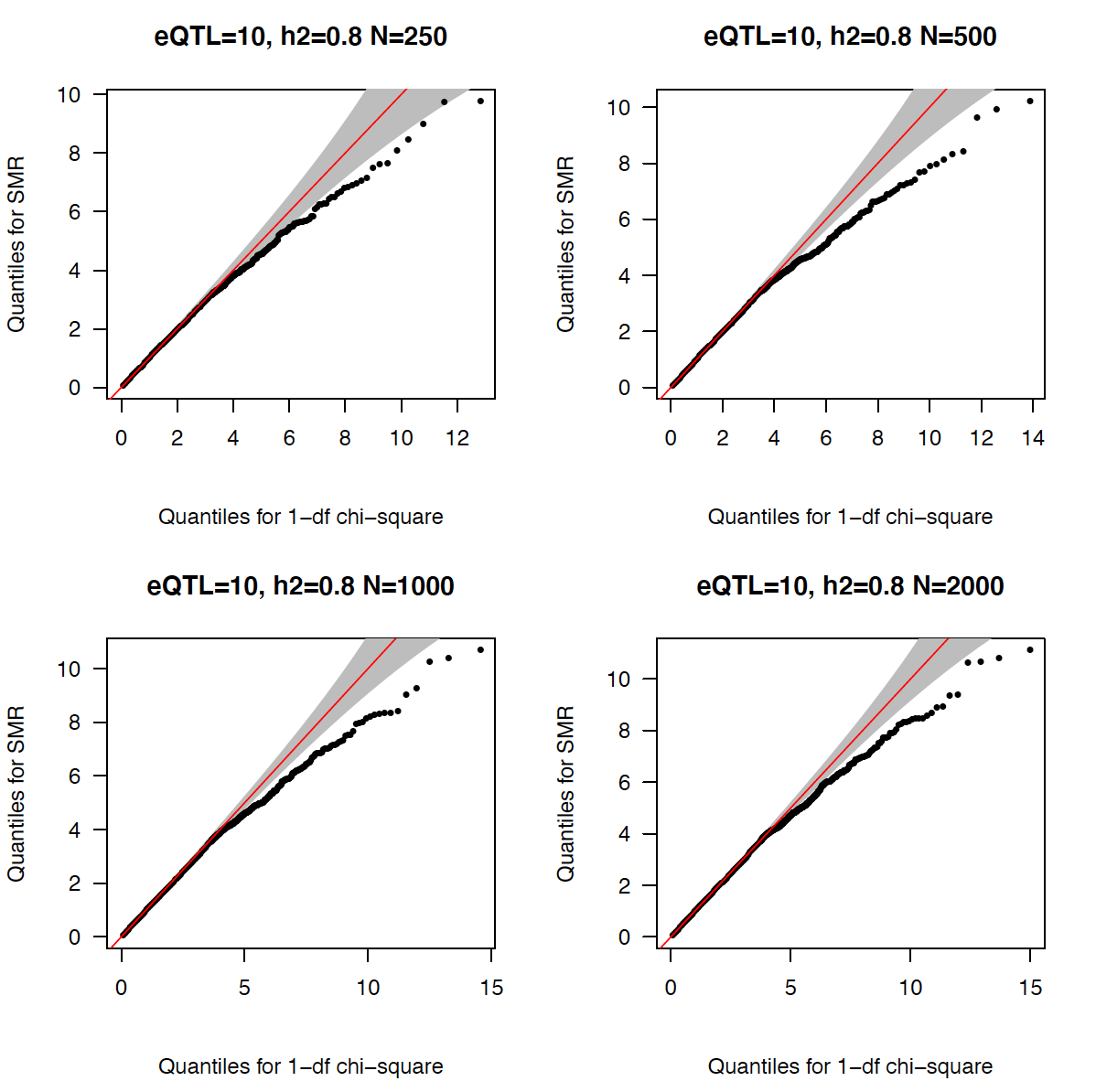


Supplementary Figure S12: Quantile-quantile plot for simulated SMR statistics against statistics of 1-df chi-squire distribution. Instrumental eQTLs for SMR test were top associated eQTL ($\boldsymbol{p<5\times}\boldsymbol{10}^{\boldsymbol{-8}}$) selected from genes whose expression levels were simulated under a genetic model in which the number of causal eQTLs is 10 (eQTL =10) and the heritability is 0.8 (h2 = 0.8) at four different sample sizes (N = 250, 500, 1000, and 2000). The grey areas represent the 95% confidence band around 1-df chi-square statistics.

Supplementary Table S1. Genes detected by test conditional on top eQTL. P-values from SMR test were also listed for comparison

| Gene | top eQTL | top eQTL chromosome | top eQTL position | top eQTL p-value | GWAS p-value | SMR p-value |
| --- | --- | --- | --- | --- | --- | --- |
| RP5-1112D6.7 | rs6568682 | 6 | 111814177 | 5.58E-09 | 3.66E-06 | 2.90E-04 |
| TSSK6 | rs4808958 | 19 | 19571752 | 7.26E-09 | 1.60E-06 | 2.22E-04 |
| GIGYF1 | rs221783 | 7 | 100292914 | 2.14E-09 | 2.23E-06 | 2.04E-04 |
| AKT3 | rs3008660 | 1 | 244024703 | 2.53E-08 | 4.76E-07 | 1.87E-04 |
| YPEL3 | rs73530179 | 16 | 30107781 | 2.21E-10 | 3.80E-06 | 1.86E-04 |
| GID4 | rs2955370 | 17 | 17966945 | 2.49E-08 | 2.73E-07 | 1.57E-04 |
| FTSJ2 | rs3757439 | 7 | 2272122 | 6.88E-11 | 3.26E-06 | 1.52E-04 |
| MARS2 | rs4286272 | 2 | 198535678 | 1.29E-09 | 1.15E-06 | 1.47E-04 |
| PCDHA3 | rs2337987 | 5 | 140188383 | 3.29E-11 | 3.31E-06 | 1.39E-04 |
| PLCL1 | rs13429970 | 2 | 198522635 | 6.47E-09 | 3.16E-07 | 1.25E-04 |
| PRSS35 | rs1170346 | 6 | 84021751 | 5.83E-10 | 8.13E-07 | 1.14E-04 |
| SDCCAG8 | rs7521079 | 1 | 243443304 | 1.08E-09 | 4.73E-07 | 1.03E-04 |
| PCNX | rs221907 | 14 | 71596347 | 2.82E-12 | 2.66E-06 | 9.69E-05 |
| RP11-380L11.3 | rs11835839 | 12 | 124431049 | 7.46E-13 | 2.92E-06 | 8.98E-05 |
| MRPS21 | rs12136332 | 1 | 150305992 | 1.57E-12 | 2.47E-06 | 8.93E-05 |
| MPPED2 | rs490937 | 11 | 30395895 | 1.45E-09 | 2.43E-07 | 8.63E-05 |
| EFTUD1P1 | rs12916198 | 15 | 84928762 | 1.08E-09 | 2.25E-07 | 7.97E-05 |
| RP11-182J1.16 | rs4842847 | 15 | 84931199 | 1.94E-09 | 1.07E-07 | 6.92E-05 |
| CLCN3 | rs4692735 | 4 | 170552632 | 6.41E-12 | 9.60E-07 | 6.59E-05 |
| RPS17 | rs783527 | 15 | 83285847 | 3.57E-08 | 4.63E-09 | 5.95E-05 |
| RGS6 | rs2190873 | 14 | 72424905 | 1.16E-08 | 1.53E-08 | 5.87E-05 |
| CDIP1 | rs9938228 | 16 | 4530597 | 5.50E-16 | 3.45E-06 | 5.67E-05 |
| AL049840.1 | rs10145755 | 14 | 104121984 | 1.64E-13 | 1.47E-06 | 5.47E-05 |
| IK | rs801174 | 5 | 140112712 | 1.19E-16 | 3.35E-06 | 5.04E-05 |
| NDUFA2 | rs1864254 | 5 | 140032947 | 8.12E-15 | 1.96E-06 | 4.98E-05 |
| CDK2AP1 | rs2695482 | 12 | 123623174 | 1.86E-08 | 1.74E-09 | 3.95E-05 |
| ASAP1 | rs4733770 | 8 | 131152665 | 1.94E-11 | 1.94E-07 | 3.91E-05 |
| CALN1 | rs2944821 | 7 | 71795998 | 5.07E-11 | 1.20E-07 | 3.79E-05 |
| ME1 | rs10455420 | 6 | 84154533 | 2.03E-17 | 2.43E-06 | 3.76E-05 |
| CUL3 | rs11685253 | 2 | 225391217 | 4.26E-09 | 5.60E-09 | 3.49E-05 |
| CYP17A1-AS1 | rs284862 | 10 | 104572081 | 1.10E-13 | 5.56E-07 | 3.30E-05 |
| CTA-250D10.23 | rs4822076 | 22 | 42364057 | 2.94E-09 | 5.34E-09 | 3.16E-05 |
| BTN3A1 | rs6907924 | 6 | 26438817 | 3.35E-09 | 4.06E-09 | 3.06E-05 |
| PTPRU | rs1498232 | 1 | 30433951 | 4.37E-08 | 5.86E-11 | 2.67E-05 |
| SFMBT1 | rs7644973 | 3 | 53082937 | 2.56E-18 | 1.49E-06 | 2.52E-05 |
| HLA-DPA1 | rs211468 | 6 | 33323265 | 1.22E-12 | 1.35E-07 | 2.29E-05 |
| WDR73 | rs12911736 | 15 | 85240008 | 7.54E-11 | 2.26E-08 | 2.21E-05 |
| RP11-380L11.4 | rs12809125 | 12 | 124407991 | 5.47E-27 | 3.08E-06 | 1.87E-05 |
| COG8 | rs12708897 | 16 | 69371927 | 2.46E-28 | 3.20E-06 | 1.78E-05 |
| ZSCAN29 | rs12905772 | 15 | 43648444 | 1.46E-30 | 3.61E-06 | 1.71E-05 |
| KCNN3 | rs11264281 | 1 | 154867221 | 4.96E-39 | 4.29E-06 | 1.45E-05 |
| ZNF184 | rs6925606 | 6 | 27357456 | 9.61E-13 | 4.29E-08 | 1.39E-05 |
| LINC01068 | rs6563116 | 13 | 80096111 | 3.30E-13 | 5.34E-08 | 1.31E-05 |
| HAPLN4 | rs7257875 | 19 | 19372150 | 5.89E-32 | 2.44E-06 | 1.21E-05 |
| NDFIP2 | rs9545078 | 13 | 79913351 | 7.17E-13 | 2.53E-08 | 1.09E-05 |
| FAM114A2 | rs1438588 | 5 | 153444950 | 2.19E-50 | 3.96E-06 | 1.05E-05 |
| NDUFA6-AS1 | rs4993393 | 22 | 42534075 | 3.81E-10 | 5.55E-10 | 1.05E-05 |
| PDF | rs12708897 | 16 | 69371927 | 4.24E-45 | 3.20E-06 | 9.76E-06 |
| ARL6IP4 | rs7957096 | 12 | 123544878 | 5.14E-09 | 1.25E-11 | 9.70E-06 |
| RP11-624D11.2 | rs1222219 | 11 | 30344345 | 4.52E-54 | 3.89E-06 | 9.69E-06 |
| ZC3H7B | rs11090045 | 22 | 41753603 | 8.55E-13 | 1.76E-08 | 9.62E-06 |
| PCDHA7 | rs13157397 | 5 | 140184442 | 1.76E-41 | 2.68E-06 | 9.21E-06 |
| U3 | rs9468297 | 6 | 28118874 | 3.42E-08 | 8.83E-14 | 9.17E-06 |
| DNAH10OS | rs12809125 | 12 | 124407991 | 2.68E-47 | 3.08E-06 | 9.04E-06 |
| MED30 | rs62521198 | 8 | 118531470 | 1.87E-25 | 9.02E-07 | 8.93E-06 |
| NEK4 | rs998909 | 3 | 52805093 | 7.63E-27 | 1.05E-06 | 8.75E-06 |
| LSM1 | rs55736052 | 8 | 38020408 | 7.20E-21 | 4.35E-07 | 8.60E-06 |
| RP11-197N18.2 | rs7957096 | 12 | 123544878 | 2.96E-09 | 1.25E-11 | 8.09E-06 |
| SNORD3B-2 | rs1969161 | 17 | 19194812 | 1.40E-37 | 1.76E-06 | 7.53E-06 |
| FSHB | rs6484478 | 11 | 30306440 | 9.89E-50 | 2.19E-06 | 6.53E-06 |
| GNL3 | rs13081155 | 3 | 52732429 | 4.51E-11 | 5.71E-10 | 6.38E-06 |
| ELAC2 | rs9908102 | 17 | 12896553 | 4.75E-55 | 2.01E-06 | 5.45E-06 |
| DCLK3 | rs75968099 | 3 | 36858583 | 1.29E-10 | 9.41E-11 | 5.06E-06 |
| PCDHA8 | rs1548699 | 5 | 140164918 | 3.04E-52 | 1.68E-06 | 4.96E-06 |
| MAPK3 | rs57149692 | 16 | 30142021 | 1.35E-34 | 8.11E-07 | 4.73E-06 |
| RP1-97D16.1 | rs9368527 | 6 | 27679445 | 9.79E-48 | 1.44E-06 | 4.70E-06 |
| RP11-282O18.3 | rs10744149 | 12 | 123708490 | 3.22E-11 | 2.42E-10 | 4.60E-06 |
| RP11-53O19.3 | rs7736952 | 5 | 44790379 | 3.92E-43 | 9.64E-07 | 3.92E-06 |
| RP5-874C20.3 | rs1736904 | 6 | 28219270 | 1.12E-08 | 3.23E-15 | 3.75E-06 |
| IP6K3 | rs570749 | 6 | 33710229 | 3.98E-14 | 4.92E-09 | 3.74E-06 |
| ALMS1 | rs1403284 | 2 | 73726662 | 1.03E-16 | 2.52E-08 | 3.73E-06 |
| ANKRD45 | rs61826793 | 1 | 173646773 | 2.53E-44 | 8.39E-07 | 3.43E-06 |
| SF3B1 | rs787999 | 2 | 198215056 | 5.20E-14 | 3.02E-09 | 3.18E-06 |
| ITIH4 | rs7620706 | 3 | 52891756 | 1.33E-21 | 9.33E-08 | 3.15E-06 |
| HIST1H4H | rs6456712 | 6 | 26302573 | 1.65E-22 | 1.11E-07 | 3.13E-06 |
| BAK1 | rs511515 | 6 | 33541507 | 1.48E-17 | 2.46E-08 | 3.06E-06 |
| ZNF391 | rs6934933 | 6 | 27354862 | 1.45E-53 | 8.42E-07 | 2.74E-06 |
| NAT8 | rs11126399 | 2 | 73613341 | 1.02E-23 | 1.01E-07 | 2.55E-06 |
| RP11-182J1.13 | rs12916198 | 15 | 84928762 | 9.02E-30 | 2.25E-07 | 2.51E-06 |
| TOM1L2 | rs12938501 | 17 | 17737258 | 1.51E-31 | 2.72E-07 | 2.51E-06 |
| XPNPEP3 | rs103197 | 22 | 41256802 | 2.68E-111 | 1.08E-06 | 1.88E-06 |
| BTN3A3 | rs6907924 | 6 | 26438817 | 3.82E-16 | 4.06E-09 | 1.88E-06 |
| RP11-490G2.2 | rs4274102 | 1 | 98416157 | 1.35E-09 | 9.43E-15 | 1.81E-06 |
| RP11-73M18.2 | rs10431750 | 14 | 104040065 | 3.07E-10 | 1.09E-13 | 1.56E-06 |
| RP11-182J1.14 | rs12916198 | 15 | 84928762 | 1.02E-45 | 2.25E-07 | 1.16E-06 |
| HIST1H4J | rs6940116 | 6 | 27708732 | 4.23E-08 | 9.05E-28 | 9.65E-07 |
| RFTN2 | rs10460394 | 2 | 198548741 | 1.39E-18 | 3.69E-09 | 9.64E-07 |
| RP11-102M11.2 | rs9825700 | 3 | 136435162 | 2.79E-14 | 1.51E-10 | 9.62E-07 |
| JRK | rs4430071 | 8 | 143737680 | 2.21E-113 | 4.90E-07 | 9.10E-07 |
| EMB | rs10076490 | 5 | 49684871 | 2.10E-67 | 2.89E-07 | 8.66E-07 |
| TSNARE1 | rs34066697 | 8 | 143352779 | 1.80E-15 | 1.94E-10 | 6.72E-07 |
| GNL3LP1 | rs159544 | 5 | 60489247 | 8.25E-16 | 2.38E-10 | 6.37E-07 |
| FAM109B | rs4822088 | 22 | 42470589 | 3.24E-24 | 9.62E-09 | 5.89E-07 |
| ZNF165 | rs172165 | 6 | 28020814 | 5.22E-11 | 2.73E-15 | 4.43E-07 |
| DDHD2 | rs9643870 | 8 | 38116792 | 3.73E-23 | 2.81E-09 | 3.50E-07 |
| BTN2A1 | rs12190859 | 6 | 27040273 | 3.73E-11 | 8.74E-16 | 3.24E-07 |
| CNNM2 | rs11191531 | 10 | 104806898 | 1.13E-10 | 5.17E-17 | 3.20E-07 |
| RP5-966M1.6 | rs4687672 | 3 | 52880543 | 3.44E-48 | 4.55E-08 | 3.05E-07 |
| ZSCAN16 | rs12173797 | 6 | 28022723 | 6.23E-13 | 2.36E-13 | 2.82E-07 |
| TYW5 | rs281791 | 2 | 200850402 | 5.32E-50 | 3.38E-08 | 2.30E-07 |
| ALMS1P | rs1403284 | 2 | 73726662 | 1.40E-44 | 2.52E-08 | 2.25E-07 |
| CSPG4P11 | rs11630760 | 15 | 85113337 | 2.10E-102 | 9.19E-08 | 2.17E-07 |
| SNAP91 | rs217295 | 6 | 84383958 | 7.03E-16 | 4.66E-12 | 1.52E-07 |
| NAGA | rs133381 | 22 | 42470608 | 3.17E-30 | 2.34E-09 | 1.20E-07 |
| ZBTB22 | rs3130271 | 6 | 33310979 | 1.31E-116 | 4.06E-08 | 9.39E-08 |
| OR2W2P | rs34466819 | 6 | 28065993 | 1.06E-14 | 1.19E-13 | 8.60E-08 |
| IQCB2P | rs34466819 | 6 | 28065993 | 1.01E-14 | 1.19E-13 | 8.51E-08 |
| CYP2D6 | rs2413670 | 22 | 42511822 | 4.51E-68 | 1.74E-08 | 8.01E-08 |
| GATAD2A | rs4808955 | 19 | 19552413 | 3.70E-19 | 1.14E-11 | 6.39E-08 |
| OR1F12 | rs9393885 | 6 | 28050009 | 2.02E-15 | 1.02E-13 | 5.71E-08 |
| SETD6 | rs246193 | 16 | 58547858 | 1.58E-30 | 7.25E-10 | 5.69E-08 |
| OR2B7P | rs9393892 | 6 | 28081394 | 1.46E-17 | 1.11E-13 | 2.12E-08 |
| THOC7 | rs832190 | 3 | 63842629 | 1.80E-37 | 4.21E-10 | 1.97E-08 |
| LINC00634 | rs6519298 | 22 | 42364997 | 4.72E-38 | 3.89E-10 | 1.80E-08 |
| ZKSCAN8 | rs1904841 | 6 | 28108085 | 9.02E-21 | 1.39E-13 | 6.67E-09 |
| AC110781.3 | rs4719319 | 7 | 1888094 | 1.06E-54 | 3.91E-10 | 6.52E-09 |
| RP1-313I6.12 | rs34466819 | 6 | 28065993 | 7.83E-21 | 1.19E-13 | 6.05E-09 |
| PCCB | rs1279949 | 3 | 135996870 | 4.92E-40 | 6.51E-11 | 4.65E-09 |
| OR2B8P | rs6934769 | 6 | 28090931 | 1.59E-31 | 7.94E-14 | 3.13E-10 |
| RP1-265C24.5 | rs2116981 | 6 | 28067951 | 8.69E-49 | 5.98E-14 | 2.35E-11 |
| ZNF192P2 | rs34024998 | 6 | 28197752 | 1.06E-71 | 1.37E-13 | 7.88E-12 |
| AL022393.7 | rs34024998 | 6 | 28197752 | 7.44E-76 | 1.37E-13 | 6.47E-12 |
| ZNF192P1 | rs4711164 | 6 | 28115156 | 2.47E-83 | 7.09E-14 | 2.96E-12 |
| ZSCAN31 | rs213236 | 6 | 28324397 | 4.69E-63 | 3.73E-15 | 1.11E-12 |
| ZKSCAN3 | rs213236 | 6 | 28324397 | 1.55E-65 | 3.73E-15 | 9.24E-13 |
| ZSCAN23 | rs13203055 | 6 | 28382410 | 4.75E-93 | 4.08E-20 | 5.17E-17 |
| BTN3A2 | rs9393704 | 6 | 26358775 | 3.14E-78 | 3.70E-26 | 3.28E-20 |
